# Supplementary material for: The Analysis, Description, and Examination of the Maize LAC Gene Family’s Reaction to Abiotic and Biotic Stress
Source: Genes (Basel). 2024 Jun 6;15(6):749. doi: 10.3390/genes15060749 (PMC11202975; doi:10.3390/genes15060749)
Supplement: Supplementary file 1 [file genes-15-00749-s001.zip › Supplementary Table S1.pdf]

Supplementary Table S1: The 39 transcripts derived from blastP search. They were re-treated in NCBI, and the cu oxidase domain was detected in SMART and Pfam websites. Which indicated that two were Cupredoxin superfamily protein, four were L-ascorbate oxidase, and seven were SKU5 similar, four were Uncharacterized protei,while only 22 were considered as laccase candidates.

Supplemental Table S1 The 39 peptide sequences derived from BlastP search

Laccase candidates	Cupredoxin superfamily protein
rna-NM_001112319.1	rna-NM_001174898.1
rna-NM_001112404.2	rna-XM_008654407.3
rna-NM_001112445.2	**L-ascorbate oxidase homologs**
rna-NM_001112451.2	rna-NM_001147615.1
rna-NM_001137204.1	rna-NM_001154125.2
rna-NM_001152419.1	rna-XM_008665289.4
rna-NM_001153186.1	rna-XM_008659156.3
rna-NM_001154470.2	**SKU5 similar**
rna-NM_001155007.2	rna-NM_001158190.1
rna-NM_001310744.1	rna-NM_001158489.1
rna-NM_001323270.1	rna-NM_001369861.1
rna-NM_001349048.1	rna-XM_008674007.2
rna-NM_001367123.2	rna-NM_001158528.2
rna-XM_008648054.4	rna-NM_001176118.1
rna-XM_008653680.3	rna-XM_008664642.3
rna-XM_008672660.2	**Uncharacterized protein**
rna-XM_008676600.3	rna-NM_001158502.1
rna-XM_020539486.1	rna-NM_001361318.1
rna-XM_020542291.3	rna-NM_001112402.2
rna-XM_020551805.3	rna-NM_001320999.1
rna-XM_020552763.3	
rna-XM_035967153.1	

Results of the best BLAST search for 22 candidate genes for maize laccase in Arabidopsis thaliana

Sequence ID	the top hits in Arabidopsis laccase genes	identity	Evalue	score	Chromosomal Location
rna-NM_001112319.1	AT3G09220.1	50.45%	1.73E-173	505	Chr3:184155708-184159486
rna-NM_001112404.2	AT5G60020.1	59.93%	0	727	Chr6:162702090-162704883
rna-NM_001112445.2	AT5G09360.1	50.55%	0	588	Chr4:186460737-186464085
rna-NM_001112451.2	AT2G29130.1	65.52%	0	764	Chr3:185593141-185595800
rna-NM_001137204.1	AT3G09220.1	43.20%	1.37E-164	481	Chr3:34243385-34245885
rna-NM_001152419.1	AT5G05390.1	59.69%	0	724	Chr1:46245888-46248623
rna-NM_001153186.1	AT5G60020.1	70.12%	0	859	Chr8:175253857-175257138
rna-NM_001154470.2	AT5G60020.1	72.22%	0	863	Chr3:185762494-185765323
rna-NM_001155007.2	AT2G40370.1	67.38%	0	796	Chr3:188759316-188761806
rna-NM_001310744.1	AT5G60020.1	72.68%	0	870	Chr3:185772524-185775771
rna-NM_001323270.1	AT3G09220.1	57.04%	0	634	Chr10:12081883-12084751
rna-NM_001349048.1	AT3G09220.1	44.97%	7.34E-164	481	Chr4:5277658-5280216
rna-NM_001367123.2	AT5G01050.1	42.78%	1.30E-138	416	Chr4:3128777-3131437
rna-XM_008648054.4	AT5G09360.1	51.23%	0	598	Chr5:216245835-216248660
rna-XM_008653680.3	AT3G09220.1	54.89%	0	601	Chr7:474562-476614
rna-XM_008672660.2	AT3G09220.1	41.13%	8.76E-156	459	Chr2:236255966-236258720
rna-XM_008676600.3	AT3G09220.1	53.99%	0	573	Chr3:184162360-184165568
rna-XM_020539486.1	AT5G60020.1	65.35%	0	799	Chr1:39861320-39864752
rna-XM_020542291.3	AT2G40370.1	64.90%	0	771	Chr8:176523561-176526424
rna-XM_020551805.3	AT3G09220.1	55.76%	1.93E-172	497	Chr4:15757576-15759632
rna-XM_020552763.3	AT5G05390.1	65.30%	0	758	Chr4:189097427-189100643
rna-XM_035967153.1	AT3G09220.1	45.94%	1.04E-168	492	Chr4:3157555-3161430
