# Supplementary material for: The Analysis, Description, and Examination of the Maize LAC Gene Family’s Reaction to Abiotic and Biotic Stress
Source: Genes (Basel). 2024 Jun 6;15(6):749. doi: 10.3390/genes15060749 (PMC11202975; doi:10.3390/genes15060749)
Supplement: Supplementary file 1 [file genes-15-00749-s001.zip › Supplementary Table S2.pdf]

Supplementary Table S2:Conserved copper ligands in L1-L4 signature sequence of 22 ZmLAC candidate genes.

Laccase		L1			L2			L3			L4	
		2 3			3 3			1 2 3			313 1 1	
ZmLAC1	88	HWHGVRQLRTGWSDGPAYVTQCPI	111	128	GTLFWHAHVSWMRATLYGAIVILPKRG	154	504	HPLHLHGF	511	566	HCHLEVHTSWGL	577
ZmLAC2	90	HWHGLRQLRNGWADGPEFVTQCPI	113	130	GTLWWHAHSSWLRATVHGALIIHPRRG	156	506	HPMHIHGY	513	568	HCHIDAHLTGGL	579
ZmLAC3	81	HWHGVKQQRNCWADGVPMVTQCPI	104	121	GTLWWHAHVFSLRGTVHGAFIIRPRRR	147	511	NPMHLHGH	518	573	HCHYDFHLSMGM	584
ZmLAC4	84	HWHGVKQRLTCWADGAGMVTQCPI	107	124	GTLWWHSHVSILRATLHGIIIIRPKSG	150	496	NPMHLHGH	503	558	HCHFEFHIAMGM	569
ZmLAC5	80	HWHGVLQLMTPWADGPSMVTQCPI	103	120	GTLWWHAHSSFLRATVYGAFIIRPRRG	146	451	HPIHLHGF	458	511	HCHLDPHVPMGL	522
ZmLAC6	80	HWHGVFQLGTPWADGPSMVTQCPI	103	120	GTLWWHAHSSLLRATVYGALIIRPSSG	146	473	HPMHLHGF	480	534	HCHIDAHLSIGL	545
ZmLAC7	96	HWHGVRQLRNGWADGPSYITQCPI	119	136	GTLWWHAHFSWLRVHLYGPLVILPKRG	162	483	HPLHLHGY	490	545	HCHFDVHLSWGL	556
ZmLAC8	87	HWHGIRQLRSGWADGPAYITQCPI	110	127	GTLWWHAHISWLRATVYGPIVILPKPG	153	483	HPLHLHGF	490	545	HCHLEVHVSWGL	556
ZmLAC9	87	HWHGIRQLRTGWADGPAYITQCPI	110	127	GTLWWHAHISWLRATVYGPLVILPKLG	153	486	HPLHLHGF	493	548	HCHLEAHTTWGL	559
ZmLAC10	85	HWHGIRQIRTGWADGPEFVTQCPI	108	125	GTLWWHAHSSWLRATVYGALIIRPREN	151	473	HPIHIHGY	480	535	HCHLDVHITWGL	546
ZmLAC11	96	HWHGVYQLLNCWNDGVPMVTQRPI	119	136	GTLWWHAHDAFLRATVYGALIIRPRNG	162	503	NPMHLHGH	510	565	HCHFEFHLAMGM	576
ZmLAC12	96	HWHGVYQLLNCWNDGVPMVTQRPI	119	136	GTLWWHAHDAFLRATVYGALIIRPRNG	162	513	NPMHLHGH	520	575	HCHFEFHLAMGM	586
ZmLAC13	105	HWHGVKQRLNCWADGVPMVTQCPI	128	145	GTLWWHAHVPCLRATLHGALIIRPRHS	171	521	NPMHLHGH	528	583	HCHFDFHLSMGM	594
ZmLAC14	1	MVTQCPI-----------------	6	24	GTLWWHAHSSMLRATVHGAIVIKPRNG	50	391	HPMHLHGH	398	453	HCHFDAHLPIGL	464
ZmLAC15	101	HWHGVDQPRNPWSDGPEYITQCPI	124	141	GTLWWHAHSEFDRATVHGAIVIHPKRG	167	491	HPMHLHGF	498	553	HCHFDRHTVWGM	564
ZmLAC16	174	HWHGVRQMRTAWADGPEFVTQCPI	197	214	GTLWWHAHSSWLRATVHGALIIRPRAG	240	567	HPIHLHGY	574	629	HCHLDVHITWGL	640
ZmLAC17	93	HWHGVDQPRNPWSDGPEYITQCPI	116	133	GTLWWHAHSDFDRATVHGAIVIHPKRG	159	483	HPMHLHGF	490	545	HCHFDRHTVWGM	556

ZmLAC18	90	HWHGVRQLRSGWADGPSYITQCPI	113	130	GTLWWHAHFSWLRATLYGPLVILPPRG	156	488	HPLHLHGY	495	550	HCHLDVHLTWGL	561
ZmLAC19	117	HWHGILQRLSCWADGPNMVSQCPI	140	158	GTLWWHAHVSFLRATVYGALILHPAAP	184	513	HPLHLHGF	520	575	HCHLDAHLPFGL	586
ZmLAC20	89	HWHGIRQLRTGWADGPAYITQCPI	112	129	GTLWWHAHISWLRATVYGPLVVLPRPG	155	485	HPLHLHGF	492	547	HCHLEVHTTWGL	558
ZmLAC21	85	HWHGIRQMRTGWADGPEFVTQCPI	108	125	GTLWWHAHSSWLRATVYGALIIRPREN	151	483	HPIHIHGY	490	545	HCHLDVHITWGL	556
ZmLAC22	90	HWHGIFQRGTPWADGPAMVTQCPV	113	130	GTLWWHAHISFLRATVYGALVLRPRAG	156	486	HPMHLHGF	493	548	HCHFDNHLDLGL	559

The equivalent signature sequences that distinguish *Zea mays* laccases include L1, HWHGX9DGX5QCPI; L2, GTLWWHAHX9GX5PX2G; L3, HPXHLHGX; L4, HLHX3HX3GX. The amino acids potentially involved in copper binding were highlighted in red, with numbers 1, 2, and 3 corresponding to Cu1, Cu2, and Cu3 ions.
