# Supplementary material for: The Analysis, Description, and Examination of the Maize LAC Gene Family’s Reaction to Abiotic and Biotic Stress
Source: Genes (Basel). 2024 Jun 6;15(6):749. doi: 10.3390/genes15060749 (PMC11202975; doi:10.3390/genes15060749)
Supplement: Supplementary file 1 [file genes-15-00749-s001.zip › Supplementary Table S3.pdf]

Supplementary Table S3. The physiochemical characteristics of 22 members in the maize LAC gene family.

**Gene Name**	**Locus Name**	**Gene ID**	**CDS Size**	**Number of Amino Acid(aa)**	**Molecular Weight(kD)**	**PI**	**Instability Index**	**Aliphatic Index**	**Grand Average of Hydropathicity**
ZmLAC1	XM_020539486.1	100193025	1812	603	65595.34	8.87	33.4	81.94	-0.008
ZmLAC2	NM_001152419.1	100279407	1824	607	65641.15	7.62	42.98	84.68	0.057
ZmLAC3	XM_008672660.2	103648173	1878	625	68726.53	5.41	55.16	76.29	-0.185
ZmLAC4	NM_001137204.1	100191779	1806	601	65716.8	6.07	36.82	79.17	-0.133
ZmLAC5	NM_001112319.1	606456	1926	641	67206.79	6.15	45.95	86.61	0.174
ZmLAC6	XM_008676600.3	103651008	1710	569	61778.17	5.61	38.84	81.95	-0.007
ZmLAC7	NM_001112451.2	732847	1749	582	63913.65	8.87	35.31	79.42	-0.144
ZmLAC8	NM_001154470.2	100281551	1749	582	63599.72	7.24	31.49	83.56	0.008
ZmLAC9	NM_001310744.1	732787	1758	585	63601.07	8.74	29.13	90	0.065
ZmLAC10	NM_001155007.2	100282094	1719	572	63190.88	5.93	36.04	84.21	-0.085
ZmLAC11	NM_001367123.2	100280281	1815	604	66315.63	6.82	38.8	81.39	-0.125
ZmLAC12	XM_035967153.1	118476850	1845	614	67450.77	6.68	41.17	80.7	-0.158
ZmLAC13	NM_001349048.1	100501262	1914	637	69380.6	5.89	41.62	88.6	0.047
ZmLAC14	XM_020551805.3	103652871	1473	490	53784.52	9.86	44.12	72.92	-0.233
ZmLAC15	NM_001112445.2	732839	1773	590	65402.34	5.8	38.53	73.86	-0.26
ZmLAC16	XM_020552763.3	103654275	2001	666	71356.8	6.78	40.4	82.97	0.038
ZmLAC17	XM_008648054.4	103627746	1749	582	64409.92	6.05	32.54	71.89	-0.281
ZmLAC18	NM_001112404.2	732786	1764	587	63818.85	9.81	37.76	83.1	-0.013
ZmLAC19	XM_008653680.3	103631856	1839	612	66147.08	6.06	32.79	86.58	0.017
ZmLAC20	NM_001153186.1	100280258	1755	584	63426.72	8.3	30.37	88.97	0.057
ZmLAC21	XM_020542291.3	100502492	1749	582	64227.02	5.94	36.5	83.59	-0.064
ZmLAC22	NM_001323270.1	103640860	1758	585	63625.04	5.7	34.57	78.21	-0.094
