# Supplementary material for: The Analysis, Description, and Examination of the Maize LAC Gene Family’s Reaction to Abiotic and Biotic Stress
Source: Genes (Basel). 2024 Jun 6;15(6):749. doi: 10.3390/genes15060749 (PMC11202975; doi:10.3390/genes15060749)
Supplement: Supplementary file 1 [file genes-15-00749-s001.zip › Supplementary Table S4.pdf]

Supplementary Table S4. Analysis of genetic replication events of the LAC family in Zea mays

Duplicated genes		Ka	Ks	Ka_Ks	Date (MY)	Duplication Type
ZmLAC5	ZmLAC6	0.397	0.729	0.545	56.48	Tandem duplication
ZmLAC8	ZmLAC9	0.150	0.307	0.490	23.63	Tandem duplication
ZmLAC1	ZmLAC7	0.259	0.599	0.432	46.06	WGD or segmental duplication
ZmLAC1	ZmLAC20	0.217	0.530	0.410	40.80	WGD or segmental duplication
ZmLAC3	ZmLAC13	0.246	0.585	0.421	45.00	WGD or segmental duplication
ZmLAC7	ZmLAC18	0.181	0.436	0.414	33.51	WGD or segmental duplication
ZmLAC7	ZmLAC20	0.265	0.490	0.541	37.69	WGD or segmental duplication
ZmLAC10	ZmLAC21	0.034	0.147	0.227	11.34	WGD or segmental duplication
ZmLAC15	ZmLAC17	0.046	0.151	0.308	11.59	WGD or segmental duplication
ZmLAC16	ZmLAC21	0.246	0.490	0.502	37.68	WGD or segmental duplication
ZmLAC18	ZmLAC20	0.268	0.476	0.562	36.64	WGD or segmental duplication
