# Supplementary material for: The Analysis, Description, and Examination of the Maize LAC Gene Family’s Reaction to Abiotic and Biotic Stress
Source: Genes (Basel). 2024 Jun 6;15(6):749. doi: 10.3390/genes15060749 (PMC11202975; doi:10.3390/genes15060749)
Supplement: Supplementary file 1 [file genes-15-00749-s001.zip › Supplementary Table S5.pdf]

**Supplementary Table S5:** Homology of LAC proteins in maize, Arabidopsis, soybean, sorghum and rice.

| **orthologous gene pairs** | |
|---|---|
| ZmLAC1 | SbLAC2 |
| ZmLAC15 | SbLAC14 |
| ZmLAC18 | SbLAC25 |
| ZmLAC19 | SbLAC3 |
| ZmLAC2 | SbLAC1 |
| ZmLAC22 | SbLAC24 |
| ZmLAC4 | SbLAC4 |
| ZmLAC5 | SbLAC11 |
| ZmLAC6 | SbLAC10 |
| ZmLAC7 | SbLAC9 |
| ZmLAC8 | SbLAC8 |
| GmLAC27 | AtLAC14 |
| GmLAC28 | AtLAC2 |
| OsLAC2 | SbLAC5 |
| OsLAC9 | SbLAC12 |

| **paralogous homologous gene pairs** | | | |
|---|---|---|---|
| AtLAC3 | AtLAC13 | GmLAC20 | GmLAC47 |
| AtLAC4 | AtLAC10 | GmLAC21 | GmLAC46 |
| AtLAC5 | AtLAC12 | GmLAC22 | GmLAC4 |
| AtLAC8 | AtLAC9 | GmLAC23 | GmLAC3 |
| ZmLAC10 | ZmLCA21 | GmLAC26 | GmLAC35 |
| ZmLAC11 | ZmLCA12 | GmLAC32 | GmLAC5 |
| ZmLAC3 | ZmLAC13 | GmLAC37 | GmLAC29 |
| ZmLAC9 | ZmLAC20 | GmLAC38 | GmLAC18 |
| OsLAC1 | OsLAC19 | GmLAC39 | GmLAC19 |
| OsLAC14 | OsLAC15 | GmLAC40 | GmLAC11 |
| OsLAC18 | OsLAC20 | GmLAC41 | GmLAC12 |
| OsLAC23 | OsLAC24 | GmLAC42 | GmLAC13 |
| OsLAC29 | OsLAC30 | GmLAC43 | GmLAC14 |
| SbLAC16 | SbLAC17 | GmLAC44 | GmLAC15 |
| SbLAC18 | SbLAC23 | GmLAC48 | GmLAC49 |
| SbLAC19 | SbLAC20 | GmLAC6 | GmLAC33 |
| | | GmLAC7 | GmLAC30 |
| | | GmLAC8 | GmLAC2 |
| | | GmLAC9 | GmLAC10 |
