# Supplementary material for: The Analysis, Description, and Examination of the Maize LAC Gene Family’s Reaction to Abiotic and Biotic Stress
Source: Genes (Basel). 2024 Jun 6;15(6):749. doi: 10.3390/genes15060749 (PMC11202975; doi:10.3390/genes15060749)

Supplementary Table S6 . The motif information of maize LAC proteins.

Motif	Sequence	Number of Amino Acids	E-value	Pfam Annotation
Motif1	YNLVDPPZRNTVAVPTGGWAAIRFVADNPGVWFMHCH LDVH	41	5.4e-617	Cu-oxidase_2 - PF07731
Motif2	SYTYRFTVTGQEGTLWWHAHSSWLRATVYGALIIRPRRG	39	1.0e-557	Cu-oxidase_3 - PF07732
Motif3	GPTIEAREGDTVVVHVVNQSPYNVTIHWHGVRQLRTGW ADGPEMVTQCPI	50	1.6e-642	Cu-oxidase_3 - PF07732
Motif4	KTYLLRLINAALNDELFFKVAGHTFTVVAADASYVKPYTT DVIVIAPGQT	50	2.7e-606	Cu-oxidase - PF00394
Motif5	KGTKVRRLRYGAVVEVVLQDTAILGAESHPMHLHGFBFF VLAQGFGNYDP	50	1.7e-589	Cu-oxidase_2 - PF07731
Motif6	YPFPKPHKEVPVJLGEWWNADVEDVIRQA	29	1.1E-282	—
Motif7	NVSDAYTINGKPGDLYNCSSK	21	1.9E-192	—
Motif8	WGLAMAFLVEDGPTPNQSLPP	21	7.6E-160	—
Motif9	RHYTFVVTMTNVTRLCKTKSITTVNGQFP	29	3.7E-223	—
Motif10	CGGPNETRFAASMNNVSFVLP	21	2.6E-140	—

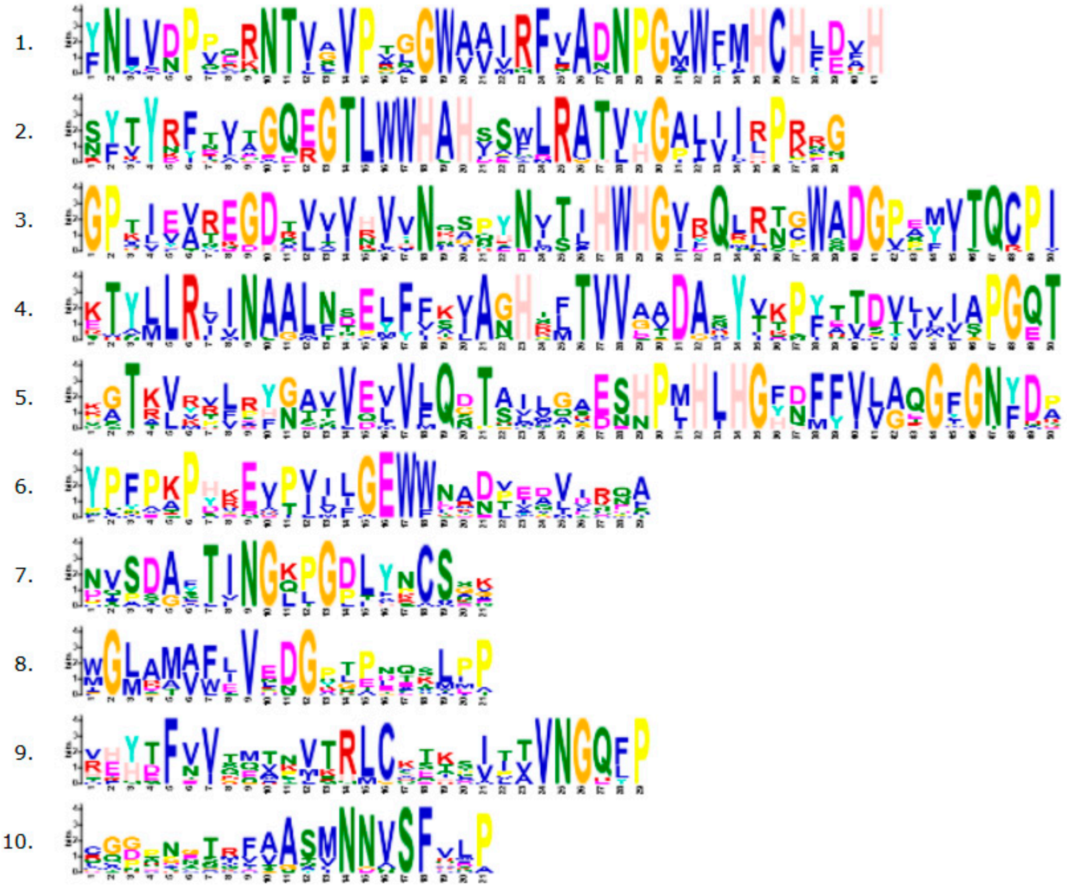

Supplement: Supplementary file 1 [file genes-15-00749-s001.zip › Supplementary Table S6.pdf]
